# Safety and efficacy of guanfacine extended-release in adults with attention-deficit/hyperactivity disorder: an open-label, long-term, phase 3 extension study

**DOI:** 10.1186/s12888-020-02867-8

**Published:** 2020-10-02

**Authors:** Akira Iwanami, Kazuhiko Saito, Masakazu Fujiwara, Daiki Okutsu, Hironobu Ichikawa

**Affiliations:** 1 Department of Psychiatry, Showa University School of Medicine, 6-11-11 Kita Karasuyama, Setagaya-ku, Tokyo, 157-8577 Japan; 2 Aiiku Counselling Office, Aiiku Research Institute, Imperial Gift Foundation Boshi-Aiiku-Kai, Tokyo, Japan; 3 Biostatistics Center, Shionogi & Co., Ltd., Osaka, Japan; 4 Clinical Research Department, Shionogi & Co., Ltd, Osaka, Japan; 5 Japan Developmental Disorders Network, Tokyo, Japan

**Keywords:** Adult, Attention deficit disorder with hyperactivity, Guanfacine, Safety, Treatment outcome

## Abstract

**Background:**

To assess the safety and efficacy of long-term administration of guanfacine extended-release (GXR) in adults with attention-deficit/hyperactivity disorder (ADHD).

**Methods:**

In this open-label, long-term, phase 3 extension study in Japan, 150 patients transitioned from a double-blind trial, and 41 newly enrolled patients received once daily GXR (starting dose 2 mg/day, maintenance dose 4–6 mg/day) for 50 weeks. Primary outcome measures were the frequency and nature of treatment-emergent adverse events (TEAEs); secondary outcome measures included the change from week 0 in ADHD Rating Scale IV with Adult Prompts (ADHD-RS-IV; Japanese version) total and subscale scores, Conners’ Adult ADHD Rating Scales (CAARS), Clinical Global Impression-Improvement (CGI-I) and Patient Global Impression-Improvement (PGI-I) scales, and quality of life (QoL) and executive functioning measures.

**Results:**

Of all patients, 94.2% (180/191) reported ≥1 TEAE and 19.9% (38/191) discontinued because of a TEAE. Most TEAEs were mild to moderate in severity; there were two serious TEAEs and no deaths. Commonly reported TEAEs (≥10% of patients) were somnolence, thirst, nasopharyngitis, decreased blood pressure, postural dizziness, bradycardia, malaise, constipation, and dizziness. Mean changes from week 0 in ADHD-RS-IV total and subscale scores and CAARS subscale scores were significantly improved in former placebo or GXR patients and new patients at last observation (*p* < .0001), and the percentage of patients with very much or much improved CGI-I and PGI-I scores increased.

**Conclusions:**

There were no major safety concerns during long-term GXR administration in adults with ADHD. After long-term treatment, patients had significant improvements from baseline in ADHD symptoms, QoL, and executive functioning.

**Trial registration:**

Japan Primary Registries Network (https://rctportal.niph.go.jp/en/): JapicCTI-163232, registered 04/21/2016.

## Background

Although attention-deficit/hyperactivity disorder (ADHD) is commonly considered a childhood disorder, it is estimated to affect up to 3% of adults worldwide [1, 2]. Adult ADHD can persist from childhood into adulthood or be newly diagnosed in adults [3] and differs from childhood ADHD in several respects. ADHD symptoms change as patients mature, with decreases in overt hyperactivity symptoms and increases in more subtle symptoms, such as inattention and disorganization [4–6]. Comorbid psychiatric and behavioral symptoms can be associated with ADHD in children and adults, which may obscure initial diagnosis of ADHD in adults [6, 7]. Nonpsychiatric comorbidities, particularly obesity, sleep disorders, and asthma, are also associated with ADHD in adults [8]. Overall, underdiagnosis and undertreatment of ADHD in adults can result in impaired quality of life (QoL) [9] and psychosocial functioning [10], addictive or risky behaviors (including substance use disorders) [6], high rates of accidental death [11], and suicide [12].

Guanfacine extended-release (GXR) is a nonstimulant, selective, α2A-adrenergic receptor agonist approved worldwide for ADHD in children and adolescents and was first approved for treatment of ADHD in adults in Japan in June 2019. As clinical trial data for the use of GXR in adults have only recently become available [13], GXR for adults was not included in a comprehensive systematic review and metaanalysis of medications for ADHD, published in 2018 [14], and is not included in current international guidelines [15]. In the first phase 3, double-blind, randomized trial conducted in adults, dose-optimized GXR treatment significantly reduced ADHD symptoms at week 10 compared with placebo, with improvements in QoL and functioning [13]. Compared with placebo, GXR was associated with an increased incidence of treatment-emergent adverse events (TEAEs) that were related to its effect on α2A-adrenergic receptors (somnolence, thirst, blood pressure decrease, postural dizziness, and constipation), but most were mild to moderate in severity and resolved during treatment [13]. Given the differences between children and adults in the clinical presentation of ADHD and associated comorbidities, assessment of the safety and efficacy of prolonged GXR treatment in adults is required.

The primary objective of this study was to assess the safety of long-term administration of once-daily GXR in adults with ADHD over 50 weeks of treatment. The secondary objective was to assess the efficacy of GXR.

## Methods

This was an open-label, long-term, phase 3 study in adults with ADHD. The study (conducted at 71 Japanese centers from December 2016 through December 2018) was approved by the following local ethics committees: Mizuo Clinic Institutional Review Board (IRB); Ehime University Hospital IRB; IHL Shinagawa East One Medical Clinic IRB; Dr. Mano Medical Clinic IRB; Odori Park Mental Clinic IRB; Tokyo Midtown Clinic IRB; Tokyo-Eki Center-Building Clinic IRB; Riverside Internal and Circulatory Medical Clinic IRB; Goryokai Hospital IRB; Himorogi Psychiatric Institute IRB; Nanko Clinic of Psychiatry IRB; Iwata Buddy’s Clinic IRB; Suzuki Internal and Circulatory Medical Clinic IRB; Kojinkai Sapporo Skin Clinic IRB; Shoda Hospital IRB; Kondo Hospital IRB; Tomisaka Clinic IRB; Yokohama Sakae Kyosai Hospital IRB; Hokkaido University Hospital IRB; Yamate Dermatoligcal Clinic IRB; Chibune General Hospital IRB; IRB of Showa University Karasuyama Hospital; Yoyogi Mental Clinic IRB; Tokai University Hospital IRB; The Jikei University Hospital IRB for Medicinal Products; Non-Profit Organization Tokyo Allergy and Respiratory Disease Research Institute IRB; Review Board of Human Rights and Ethics for Clinical Studies; Nara Medical University Hospital IRB; University of Fukui Hospital IRB; Hayashi Diabetes Clinic IRB, and conducted in accordance with Good Clinical Practice and the Declaration of Helsinki. All patients provided written informed consent before participating in the study. The previous double-blind trial (DBT) [13] and this study were registered at the Japan Primary Registries Network (JapicCTI-163231).

### Study population

Newly enrolled patients and the patients who completed the previous DBT and who consented to transition to this open-label study were eligible for inclusion. The main inclusion criteria for new patients were adult men or women (age ≥18 years) with a diagnosis of ADHD (*Diagnostic and Statistical Manual of Mental Disorders* (Fifth Edition) [*DSM-5*]) [16], ADHD Rating Scale IV with Adult Prompts (ADHD-RS-IV; Japanese version) total score ≥24, and a Clinical Global Impression-Severity of Illness (CGI-S) scale score ≥4. Exclusion criteria were reported in detail previously [13]. In brief, the main exclusion criteria were a diagnosed or documented moderate/severe psychiatric disorder (based on *DSM-5*) requiring drug treatment, a history of substance use disorder or seizures, persons considered at risk of suicide, a history or evidence of cardiovascular disease, and use of medications affecting blood pressure or heart rate.

### Study design

This open-label study was dose optimized and noncontrolled and comprised a 50-week treatment period, a 2-week tapered dose-reduction period, and a 1-week follow-up period (Additional file 1). All patients received a single dose of GXR once daily at approximately the same time (morning or afternoon), starting at a minimum dose of 2 mg/day and increasing to a maintenance dose of 4–6 mg/day for 50 weeks. Forced dose increments of 1-mg increases up to a total of 4 mg, followed by 1-mg increases or reductions at ≥5-day intervals to maintain the dose between 4 and 6 mg, were allowed at the investigator’s discretion for patients with no safety concerns and CGI-S scores ≥3. During the tapered dose-reduction period, doses were decreased by 1 mg at ≥3-day intervals over 2 weeks.

### Outcome measures

Safety measures included the type and frequency of TEAEs (Medical Dictionary for Regulatory Activities, v19.0) and vital signs at each visit, and electrocardiogram (ECG) parameters and clinical laboratory tests (weeks 0, 10, 22, 34, 50, and study discontinuation).

Efficacy outcomes included physician-rated measures (ADHD-RS-IV total and subscale scores, Conners’ Adult ADHD Rating Scales [CAARS], and CGI-Improvement [CGI-I] and CGI-S scales) [17–20] and patient-rated measures (Patient Global Impression-Improvement [PGI-I] scale, the Adult ADHD Quality of Life Questionnaire [AAQoL], and the Behavior Rating Inventory of Executive Function-Adult Version [BRIEF-A]) [19, 21–23]. ADHD-RS-IV and CGI-S were assessed at each visit from weeks 0–50 or discontinuation. CGI-I and PGI-I were assessed at each visit from weeks 1–50 or discontinuation, CAARS was assessed at weeks 0, 22, and 50 or discontinuation, and AAQoL and BRIEF-A were assessed at weeks 0, 10, 22, 34, and 50 or discontinuation.

### Statistical analysis

The target sample size was 190 patients to allow for 100 patients completing 1 year of treatment. All patients who received at least one dose of GXR were included in the analyses. All TEAEs between the first intake of study drug and follow-up observation were analyzed. For analyses of ADHD-RS-IV total and subscale scores, CAARS scores, AAQoL scores, and BRIEF-A, mean (95% confidence intervals [CIs]) at each visit were reported. Mean differences in scores from week 0 (screening period) were assessed at each visit using two-sided *t* tests for ADHD-RS-IV total and subscale scores, CAARS scores, and AAQoL scores. Illness severity and improvement (CGI-S, CGI-I, or PGI-I) rates at each visit from week 0 were assessed using the Clopper–Pearson method. Missing data were not imputed for efficacy analyses; statistical analyses were performed using SAS Version 9.2 or higher (SAS Institute Inc., Cary, NC, USA).

## Results

### Patient disposition and baseline characteristics

**Fig. 1:**
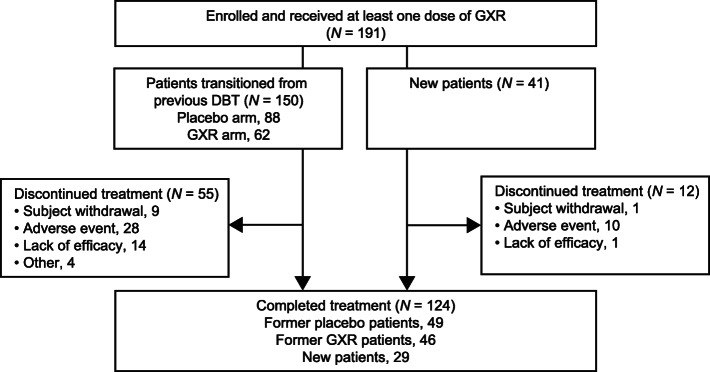
Patient disposition. DBT: double-blind trial; GXR, guanfacine extended-release

A total of 191 patients were enrolled, received at least one dose of study drug, and were included in the analyses (Fig. 1); 150 had transitioned from the previous DBT (former placebo or GXR patients) and 41 were newly enrolled (new patients). Of the enrolled patients, 124 (95 transitioned, 29 new) completed the study. The main reason for discontinuation was adverse events from all populations (Fig. 1).

During the study, mean (standard deviation) treatment duration was 254.9 (136.5) days for all patients (transitioned: 247.6 [140.1]; new: 281.7 [120.3]), and the most frequently taken doses of GXR were 6 mg (38% of patients), 4 mg (35% of patients), and 5 mg (17% of patients).

In all patients, approximately half had combined presentation or predominantly inattentive presentation, and approximately half had been treated with ADHD medication previously (Table 1). At the start of the DBT for those who transitioned and at the start of long-term treatment for new patients, mean ADHD-RS-IV total scores were approximately 32 among all patients, but there was a higher proportion of new patients (70.7%) with ADHD-RS-IV total scores ≥30 than former placebo (51.6%) or GXR (53.4%) patients.

**Table 1:** Patient Demographics and Baseline Characteristics

Characteristic	Former placebo patients^a^ (*N* = 88)	Former GXR patients^a^ (*N* = 62)	New patients (*N* = 41)	All patients (*N* = 191)
Male, n (%)	56 (63.6)	47 (75.8)	27 (65.9)	130 (68.1)
Age, y, mean (SD)	34.2 (10.1)	30.6 (8.2)	34.3 (9.2)	33.1 (9.4)
<30 y, n (%)	32 (36.4)	32 (51.6)	15 (36.6)	79 (41.4)
30 to <40 y, n (%)	31 (35.2)	18 (29.0)	13 (31.7)	62 (32.5)
≥40 y, n (%)	25 (28.4)	12 (19.4)	13 (31.7)	50 (26.2)
Previous disease recorded in medical history, yes, n (%)	46 (52.3)	29 (46.8)	16 (39.0)	91 (47.6)
Prior ADHD medication^b^, yes, n (%)	42 (47.7)	30 (48.4)	19 (46.3)	91 (47.6)
Atomoxetine	24 (27.3)	15 (24.2)	11 (26.8)	50 (26.2)
Methylphenidate	22 (25.0)	15 (24.2)	10 (24.4)	47 (24.6)
Other	0	3 (4.8)	0	3 (1.6)
ADHD presentation, n (%)				
Combined presentation	46 (52.3)	31 (50.0)	20 (48.8)	97 (50.8)
Predominantly inattentive presentation	40 (45.5)	29 (46.8)	21 (51.2)	90 (47.1)
Predominantly hyperactive-impulsive presentation	2 (2.3)	2 (3.2)	0	4 (2.1)
Baseline ADHD-RS-IV total score, mean (SD)	32 (7.1)	31.7 (6.0)	32.8 (5.9)	32.1 (6.5)
<30, n (%)	41 (46.6)	30 (48.4)	12 (29.3)	83 (42.5)
≥30, n (%)	47 (53.4)	32 (51.6)	29 (70.7)	108 (56.5)

*ADHD* Attention-deficit/hyperactivity disorder, *ADHD-RS-IV* Attention-Deficit/Hyperactivity Disorder Rating Scale IV with Adult Prompts, *DBT* Double-blind trial, *GXR* Guanfacine extended-release, *SD* Standard deviation

^a^ Baseline data are at enrollment in the previous DBT

^b^ Patients might have more than one prior ADHD medication

### Safety and tolerability

In general, no new or unexpected adverse events were reported during long-term treatment (Table 2). A total of 830 TEAEs were reported by 180 patients (94.2%), with most considered to be drug related (83.8% of all patients). Most TEAEs were mild to moderate in severity, and no deaths were reported (Table 2). Compared with former placebo patients and new patients, a smaller proportion of former GXR patients experienced treatment-related TEAEs or moderate severity TEAEs or discontinued because of a TEAE (Table 2).

**Table 2:** Patients Experiencing TEAEs During Long-term Treatment With GXR

Variable	Former placebo patients (*N* = 88)	Former GXR patients (*N* = 62)	New patients (*N* = 41)	All patients (*N* = 191)
All TEAEs, n (%)	82 (93.2)	58 (93.5)	40 (97.6)	180 (94.2)
Related	74 (84.1)	49 (79.0)	37 (90.2)	160 (83.8)
Mild^a^	46 (52.3)	39 (62.9)	20 (48.8)	105 (50.0)
Moderate^a^	35 (39.8)	17 (27.4)	20 (48.8)	72 (37.7)
Severe^a^	1 (1.1)	2 (3.2)	0	3 (1.6)
Leading to study discontinuation, n (%)	22 (25.0)	6 (9.7)	10 (24.4)	38 (19.9)
Serious TEAEs, n (%)	0	1 (1.6)	1 (2.4)	2 (1.0)
Death, n (%)	0	0	0	0
TEAEs occurring in ≥10% of all patients, n (%)				
Somnolence	34 (38.6)	27 (43.5)	19 (46.3)	80 (41.9)
Thirst	34 (38.6)	13 (21.0)	12 (29.3)	59 (30.9)
Nasopharyngitis	19 (21.6)	20 (32.3)	14 (34.1)	53 (27.7)
Blood pressure decrease	16 (18.2)	11 (17.7)	11 (26.8)	38 (19.9)
Postural dizziness	18 (20.5)	8 (12.9)	10 (24.4)	36 (18.8)
Bradycardia	16 (18.2)	10 (16.1)	7 (17.1)	33 (17.3)
Malaise	17 (19.3)	6 (9.7)	7 (17.1)	30 (15.7)
Constipation	8 (9.1)	7 (11.3)	6 (14.6)	21 (11.0)
Dizziness	13 (14.8)	5 (8.1)	2 (4.9)	20 (10.5)

*GXR* Guanfacine extended-release, *TEAE* Treatment-emergent adverse event

^a^ For patients experiencing the same coded event more than once, the severest category was reported

Two patients experienced a serious TEAE. One continuing patient was diagnosed with acute myeloid leukemia 380 days after starting treatment (81 days after completing the tapering period), which was considered unrelated to study drug. One new patient, with a preexisting condition requiring prescription of verapamil, experienced supraventricular tachycardia of moderate severity 255 days after starting GXR; the patient recovered following treatment and discontinuation of GXR.

The most commonly reported TEAEs (incidence ≥10%) in all patients were somnolence, thirst, nasopharyngitis, decreased blood pressure, postural dizziness, bradycardia, malaise, constipation, and dizziness (Table 2). Except for nasopharyngitis, most events were considered related to GXR. Study drug discontinuation because of TEAEs was reported for 19.9% of all patients (Table 2). The main TEAEs resulting in GXR discontinuation were somnolence (nine patients), blood pressure reduction (eight patients), malaise (six patients), bradycardia (four patients), and postural dizziness (three patients) or dizziness (three patients). All events resulting in GXR discontinuation were of mild or moderate severity except for one event of severe bradycardia, which occurred 70 days after commencing treatment. The GXR dose at onset was 6 mg. The patient discontinued GXR and recovered without treatment.

There were no clinically relevant changes in blood pressure, pulse rate, or ECG parameters (Table 3) or clinical laboratory tests after 50 weeks of treatment with GXR. For all patients, the mean change from week 0 in systolic blood pressure and diastolic blood pressure between week 1 and week 50 ranged from −9.54 to −3.82 mmHg and from −8.37 to −2.93 mmHg, respectively; the mean change in pulse rate ranged from −9.04 to −2.12 beats/minute; and the mean change in body weight between week 4 and week 50 ranged from −0.33 to 0.28 kg. For all patients, small changes in ECG parameters were observed during long-term treatment, which gradually recovered to the levels observed at week 0 by the end of treatment (weeks 50–52). The mean change from week 0 at last observation in the treatment period was a decrease in heart rate of 6.75 beats/minute, an increase in RR interval of 115.12 msec, an increase in PR interval of 3.55 msec, an increase in QT interval of 12.96 msec, and a decrease in QTc corrected by Bazett’s formula (QTcB) interval of 9.91 msec and a decrease in QTc corrected by Fridericia’s formula (QTcF) interval of 2.36 msec. The changes in QRS interval were variable during long-term treatment.

**Table 3:** Change in Body Weight and Cardiovascular Parameters During Long-term Treatment With GXR

Parameter	Patient population	Week 0 Mean (SD)	Mean (SD) change from week 0 at last observation in the treatment period
Body weight, kg	Former placebo patients	66.89 (15.1)	0.12 (2.8)
	Former GXR patients	67.34 (12.7)	−0.34 (4.4)
	New patients	68.40 (15.2)	−0.34 (3.4)
Pulse rate, bpm	Former placebo patients	73.56 (8.5)	−5.60 (12.4)
	Former GXR patients	72.94 (11.7)	−5.48 (9.8)
	New patients	77.22 (11.7)	−6.74 (11.8)
Systolic BP, mmHg	Former placebo patients	115.39 (14.2)	−6.19 (11.9)
	Former GXR patients	117.58 (13.2)	−7.31 (11.7)
	New patients	119.54 (16.9)	−8.27 (14.7)
Diastolic BP, mmHg	Former placebo patients	72.38 (10.5)	−4.11 (10.1)
	Former GXR patients	74.51 (11.2)	−6.88 (10.6)
	New patients	75.06 (14.0)	−6.52 (10.8)
ECG parameters			
Heart rate, bpm	Former placebo patients	65.7 (8.5)	−7.9 (10.0)
	Former GXR patients	63.5 (9.9)	−3.8 (9.5)
	New patients	69.30 (9.4)	−8.70 (12.4)
RR interval, msec	Former placebo patients	926.0 (114.2)	135.7 (165.3)
	Former GXR patients	964.6 (147.3)	72.9 (161.8)
	New patients	881.55 (134.5)	134.30 (192.9)
PR interval, msec	Former placebo patients	153.1 (20.5)	3.1 (12.0)
	Former GXR patients	149.2 (19.5)	4.2 (11.6)
	New patients	150.87 (21.0)	3.44 (15.3)
QRS interval, msec	Former placebo patients	98.6 (10.4)	0.5 (6.5)
	Former GXR patients	100.7 (16.0)	−0.1 (6.4)
	New patients	98.58 (7.9)	0.58 (5.7)
QT interval, msec	Former placebo patients	396.9 (29.0)	16.9 (25.9)
	Former GXR patients	403.5 (24.3)	4.6 (25.6)
	New patients	387.86 (28.3)	17.05 (27.3)
QTcB, msec	Former placebo patients	413.5 (23.3)	−9.9 (18.1)
	Former GXR patients	413.1 (21.9)	− 9.5 (16.8)
	New patients	414.85 (21.4)	−10.56 (21.4)
QTcF, msec	Former placebo patients	407.8 (22.5)	−1.0 (14.4)
	Former GXR patients	409.7 (17.0)	−5.0 (13.4)
	New patients	405.41 (19.8)	−1.21 (14.8)

*BP* Blood pressure, *bpm* Beats per minute, *ECG* Electrocardiogram, *GXR* Guanfacine extended-release, *QTcB* QTc corrected by Bazett’s formula, *QTcF* QTc corrected by Fridericia’s formula, *SD* Standard deviation

### Efficacy

#### ADHD-RS-IV

**Fig. 2:**
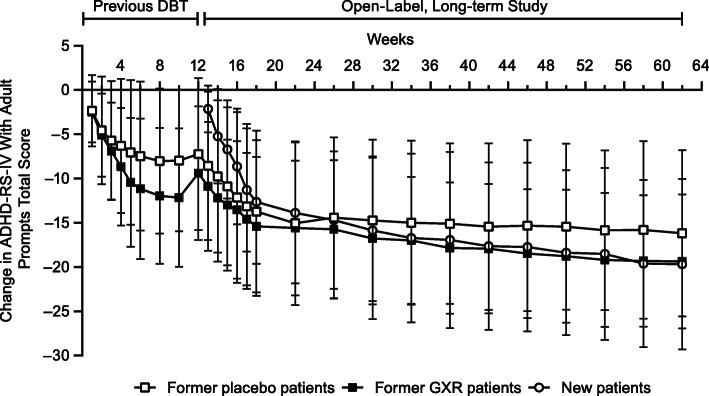
Change from baseline in ADHD-RS-IV total scores. Data are the mean change from baseline (i.e., the start of the previous double-blind trial [DBT]) for patients who transitioned from the placebo arm and guanfacine extended-release (GXR) arm and the mean change from week 0 of the long-term treatment study for new patients. Error bars denote standard deviations. ADHD-RS-IV: Attention-Deficit/Hyperactivity Disorder Rating Scale IV with Adult Prompts

Significant improvements in ADHD symptoms were reported in all patient populations during long-term treatment with GXR (Table 4). ADHD-RS-IV total and subscale scores significantly decreased (improved) compared with week 0 up to last observation and week 50 (Table 4; all *p* < .0001). The mean (95% CI) ADHD-RS-IV total scores at last observation were 18.82 (16.47, 21.16) for former placebo patients, 14.44 (12.08, 16.79) for former GXR patients, and 16.27 (13.21, 19.32) for new patients. Rapid improvements in ADHD-RS-IV total scores were observed within the first 1–6 weeks of long-term treatment, which were sustained up to week 50 for all populations (Fig. 2).

**Table 4:** Key Efficacy Measures During Long-term Treatment With GXR

			Week 50		Last observation in the treatment period	
Endpoint	Patient populations	Week 0	Change from week 0	*p*-value	Change from week 0	*p*-value
ADHD-RS-IV^a^, mean (95% CI)						
Total scores	Former placebo patients	24.76 (22.53, 26.99)	−8.31 (−10.72, −5.89)	<.0001	−5.94 (−7.53, −4.36)	<.0001
	Former GXR patients	22.31 (19.65, 24.97)	−9.11 (−11.19, −7.03)	<.0001	−7.87 (−9.68, −6.06)	<.0001
	New patients	32.80 (30.93, 34.68)	−19.69 (−23.35, −16.03)	<.0001	−16.54 (−19.77, −13.31)	<.0001
Inattention score	Former placebo patients	17.36 (15.97, 18.76)	−5.51 (−7.15, −3.87)	<.0001	−3.90 (−5.03, −2.76)	<.0001
	Former GXR patients	15.37 (13.68, 17.07)	−5.82 (−7.22, −4.42)	<.0001	−4.87 (−6.04, −3.70)	<.0001
	New patients	21.68 (20.12, 23.24)	−12.10 (−14.70, −9.51)	<.0001	−10.02 (−12.28, −7.76)	<.0001
Hyperactivity-impulsivity score	Former placebo patients	7.40 (6.15, 8.65)	−2.80 (−4.00, −1.59)	<.0001	−2.05 (−2.77, −1.32)	<.0001
	Former GXR patients	6.94 (5.55, 8.32)	−3.29 (−4.40, −2.17)	<.0001	−3.00 (−3.96, −2.04)	<.0001
	New patients	11.12 (9.50, 12.74)	−7.59 (−9.81, −5.36)	<.0001	−6.51 (−8.25, −4.78)	<.0001
CAARS scores (*DSM-IV*)^a^, mean (95% CI)						
Total ADHD symptoms	Former placebo patients	25.08 (22.93, 27.23)	−6.27 (−8.65, −3.89)	<.0001	−4.60 (−6.17, −3.02)	<.0001
	Former GXR patients	22.74 (20.07, 25.42)	−8.38 (−10.90, −5.86)	<.0001	−7.30 (−9.49, −5.10)	<.0001
	New patients	31.32 (28.64, 33.99)	−17.31 (−20.89, −13.73)	<.0001	−15.08 (−18.49, −11.66)	<.0001
Inattentive symptoms	Former placebo patients	17.40 (16.14, 18.65)	−3.96 (−5.57, −2.35)	<.0001	−2.90 (−4.02, −1.79)	<.0001
	Former GXR patients	15.55 (13.90, 17.19)	−5.40 (−7.03, −3.77)	<.0001	−4.51 (−5.89, −3.13)	<.0001
	New patients	20.39 (18.48, 22.30)	−11.00 (−13.54, −8.46)	<.0001	−9.15 (−11.47, −6.83)	<.0001
Hyperactive- impulsive symptoms	Former placebo patients	7.68 (6.38, 8.98)	−2.31 (−3.55, −1.06)	.0005	−1.69 (−2.47, −0.92)	<.0001
	Former GXR patients	7.19 (5.79, 8.60)	−2.98 (−4.30, −1.65)	<.0001	−2.79 (−3.92, −1.66)	<.0001
	New patients	10.93 (9.36, 12.49)	−6.31 (−8.17, −4.45)	<.0001	−5.93 (−7.56, −4.29)	<.0001
CGI-I response rates^b^, % of patients (95% CI)						
Improvement rate (disease scores 1 or 2)	Former placebo patients	3.4 (0.7, 9.6)^c^	51.0 (36.3, 65.6)	NA	35.2 (25.3, 46.1)	NA
	Former GXR patients	4.8 (1.0, 13.5)^c^	64.4 (48.8, 78.1)	NA	53.2 (40.1, 66.0)	NA
	New patients	0.0 (0.0, 8.6)^c^	79.3 (60.3, 92.0)	NA	65.9 (49.4, 79.9)	NA
PGI-I response rates^b^, % of patients (95% CI)						
Improvement rate (disease scores 1 or 2)	Former placebo patients	8.0 (3.3, 15.7)^c^	28.6 (16.6, 43.3)	NA	19.3 (11.7, 29.1)	NA
	Former GXR patients	9.7 (3.6, 19.9)^c^	42.2 (27.7, 57.8)	NA	33.9 (22.3, 47.0)	NA
	New patients	9.8 (2.7, 23.1)^c^	37.9 (20.7, 57.5)	NA	31.7 (18.1, 48.1)	NA
Patients not ill or borderline mentally ill^b^, % of patients (95% CI)						
CGI-S scores 1 or 2	Former placebo patients	0.0 (0.0, 4.1)	14.3 (5.9, 27.2)	NA	8.0 (3.3, 15.7)	NA
	Former GXR patients	0.0 (0.0, 5.8)	26.7 (14.6, 41.9)	NA	22.6 (12.9, 35.0)	NA
	New patients	0.0 (0.0, 8.6)	20.7 (8.0, 39.7)	NA	17.1 (7.2, 32.1)	NA
AAQoL^a^, mean (95% CI)						
Total score	Former placebo patients	46.43 (43.21, 49.64)	4.13 (0.50, 7.75)	.0266	2.81 (0.31, 5.30)	.0282
	Former GXR patients	54.27 (49.78, 58.77)	4.29 (0.35, 8.23)	.0334	4.04 (0.88, 7.20)	.0131
	New patients	43.28 (38.38, 48.17)	12.75 (6.68, 18.81)	.0002	9.22 (4.11, 14.34)	.0008
Life productivity	Former placebo patients	48.04 (43.75, 52.33)	2.64 (−3.32, 8.61)	.3775	2.89 (−0.94, 6.72)	.1377
	Former GXR patients	57.88 (52.69, 63.08)	8.74 (4.69, 12.79)	<.0001	8.08 (4.76, 11.41)	<.0001
	New patients	44.29 (37.72, 50.86)	17.08 (9.11, 25.06)	.0001	14.38 (7.75, 21.00)	<.0001
Psychological health	Former placebo patients	47.02 (42.20, 51.83)	5.27 (0.57, 9.97)	.0286	2.60 (−1.14, 6.34)	.1710
	Former GXR patients	54.91 (48.88, 60.93)	2.78 (−2.69, 8.25)	.3117	1.57 (−2.82, 5.96)	.4771
	New patients	43.39 (36.92, 49.86)	11.35 (4.29, 18.41)	.0027	5.62 (−0.57, 11.82)	.0739
Life outlook	Former placebo patients	41.93 (38.20, 45.66)	2.59 (−1.97, 7.15)	.2597	1.37 (−1.76, 4.51)	.3868
	Former GXR patients	46.10 (41.64, 50.56)	−1.90 (−6.44, 2.63)	.4016	−0.35 (−4.08, 3.37)	.8510
	New patients	40.17 (35.10, 45.24)	8.23 (1.25, 15.21)	.0225	6.06 (0.71, 11.41)	.0275
Relationships	Former placebo patients	48.47 (44.01, 52.92)	8.16 (2.70, 13.63)	.0042	4.88 (1.15, 8.61)	.0109
	Former GXR patients	57.02 (50.97, 63.06)	5.00 (−1.10, 11.10)	.1058	4.26 (−0.82, 9.34)	.0984
	New patients	45.24 (38.72, 51.77)	11.21 (3.26, 19.16)	.0074	6.63 (−0.12, 13.37)	.0542
Inhibit	Former placebo patients	57.24 (54.81, 59.66)	−3.69 (−6.39, −0.99)	.0084	−2.39 (−4.15, −0.63)	.0084
	Former GXR patients	51.68 (49.20, 54.16)	−1.84 (−4.04, 0.35)	.0977	−2.66 (−4.65, −0.66)	.0098
	New patients	59.68 (56.49, 62.87)	−8.07 (−11.76, −4.38)	.0001	−8.25 (−11.28, −5.22)	<.0001
Shift	Former placebo patients	69.55 (66.83, 72.27)	−5.84 (−9.00, −2.68)	.0005	−3.29 (−5.51, −1.08)	.0040
	Former GXR patients	62.73 (59.04, 66.41)	−4.60 (−8.30, −0.90)	.0159	−3.70 (−6.57, −0.84)	.0121
	New patients	70.07 (66.33, 73.81)	−8.86 (−11.81, −5.91)	<.0001	−8.63 (−11.18, −6.07)	<.0001
Emotional control	Former placebo patients	57.80 (55.51, 60.08)	−4.39 (−7.55, −1.23)	.0075	−3.26 (−5.34, −1.18)	.0025
	Former GXR patients	53.32 (50.75, 55.89)	−2.22 (−4.97, 0.53)	.1104	−1.52 (−3.72, 0.67)	.1703
	New patients	59.98 (56.74, 63.22)	−5.41 (−7.60, −3.23)	<.0001	−4.53 (−6.68, −2.37)	.0001
Self-monitor	Former placebo patients	61.81 (58.70, 64.91)	−6.39 (−8.98, −3.79)	<.0001	−4.48 (−6.45, −2.52)	<.0001
	Former GXR patients	56.06 (52.73, 59.40)	−4.93 (−8.10, −1.76)	.0031	−4.23 (−6.87, −1.58)	.0022
	New patients	61.24 (56.70, 65.79)	−7.86 (−11.77, − 3.96)	.0003	−6.23 (−10.05, −2.40)	.0021
Behavioral regulation index	Former placebo patients	63.09 (60.46, 65.73)	−6.02 (−9.06, −2.98)	.0002	−4.06 (−6.05, −2.07)	.0001
	Former GXR patients	56.39 (53.43, 59.35)	−3.71 (−6.52, −0.90)	.0109	−3.26 (−5.52, − 1.01)	.0053
	New patients	64.73 (61.24, 68.23)	−8.72 (−11.53, −5.92)	<.0001	−7.98 (−10.72, −5.23)	<.0001
Initiate	Former placebo patients	68.51 (65.65, 71.38)	−5.49 (−8.51, −2.46)	.0006	−3.87 (−5.94, −1.80)	.0004
	Former GXR patients	59.71 (56.58, 62.84)	−3.31 (−6.64, 0.02)	.0514	−2.03 (−4.87, 0.80)	.1563
	New patients	69.10 (64.97, 73.22)	−10.38 (−13.98, −6.78)	<.0001	−8.95 (−12.11, −5.79)	<.0001
Working memory	Former placebo patients	73.91 (71.07, 76.75)	−4.80 (−8.45, −1.15)	.0111	−3.31 (−5.68, −0.93)	.0069
	Former GXR patients	66.18 (62.57, 69.78)	−4.78 (−8.24, −1.32)	.0079	−3.92 (−6.70, −1.13)	.0066
	New patients	74.10 (70.29, 77.91)	−10.93 (−14.98, −6.88)	<.0001	−10.15 (−13.73, −6.57)	<.0001
Plan/organize	Former placebo patients	70.51 (67.74, 73.28)	−3.86 (−6.94, −0.78)	.0152	−2.32 (−4.37, −0.27)	.0270
	Former GXR patients	63.02 (59.52, 66.51)	−3.69 (−7.09, −0.29)	.0340	−3.00 (−5.90, −0.10)	.0426
	New patients	70.51 (66.57, 74.45)	−8.90 (−12.63, −5.16)	<.0001	−8.75 (−11.90, −5.60)	<.0001
Task monitor	Former placebo patients	72.63 (69.56, 75.69)	−6.76 (−9.95, −3.56)	<.0001	−4.07 (−6.35, −1.79)	.0006
	Former GXR patients	63.85 (60.22, 67.49)	−7.02 (−11.06, −2.99)	.0011	−4.89 (−8.34, −1.43)	.0063
	New patients	70.71 (66.07, 75.34)	−8.93 (−13.17, −4.70)	.0002	−8.35 (−12.12, −4.58)	<.0001
Organization of materials	Former placebo patients	65.97 (63.95, 67.98)	−5.00 (−7.82, −2.18)	.0008	−3.24 (−5.21, −1.26)	.0016
	Former GXR patients	58.61 (55.77, 61.45)	−3.31 (−5.55, −1.08)	.0046	−2.87 (−4.62, −1.11)	.0018
	New patients	65.73 (62.43, 69.03)	−8.41 (−11.90, −4.93)	<.0001	−7.88 (−11.03, −4.72)	<.0001
Metacognition index	Former placebo patients	73.36 (70.51, 76.21)	−5.80 (−9.04, −2.55)	.0008	−3.75 (−5.86, −1.65)	.0007
	Former GXR patients	64.16 (60.65, 67.68)	−5.02 (−8.21, −1.83)	.0028	−3.82 (−6.44, −1.20)	.0050
	New patients	73.24 (69.29, 77.20)	−11.14 (−14.95, −7.33)	<.0001	−10.35 (−13.69, −7.01)	<.0001
GEC index	Former placebo patients	70.52 (67.76, 73.29)	−6.41 (−9.59, −3.22)	.0002	−4.22 (−6.30, −2.15)	.0001
	Former GXR patients	61.73 (58.32, 65.13)	−4.84 (−7.96, −1.73)	.0031	−3.90 (−6.45, −1.36)	.0032
	New patients	71.10 (67.26, 74.93)	−10.86 (−14.29, −7.43)	<.0001	−10.05 (−13.21, −6.89)	<.0001

*AAQoL* Adult ADHD Quality of Life Questionnaire, *ADHD-RS-IV* Attention-Deficit/Hyperactivity Disorder Rating Scale IV with Adult Prompts, *BRIEF-A* Behavior Rating Inventory of Executive Function-Adult Version, *CAARS* Conners’ Adult ADHD Rating Scales, *CGI-I* Clinical Global Impression-Improvement, *CGI-S* Clinical Global Impression-Severity of Illness, *CI* Confidence interval, *DSM-IV Diagnostic and Statistical Manual of Mental Disorders* (Fourth Edition), *GEC* Global Executive Composite, *GXR* Guanfacine extended-release, *NA* Not applicable, *PGI-I* Patient Global Impression-Improvement

^a^ Change from start of long-term treatment calculated using week 50 or last observation in the treatment period and assessed using two-sided *t* tests

^b^ Data are response rates at each time point. Differences in response rates from the start of long-term treatment or week 1 and week 50 or last observation in the treatment period were assessed using two-sided *t* tests

^c^ Data are response rates at week 1 of long-term treatment

#### CAARS

The mean (95% CI) CAARS total scores at last observation were 20.61 (18.27, 22.95) for former placebo patients, 15.66 (13.20, 18.11) for former GXR patients, and 16.68 (13.84, 19.51) for new patients. In addition, there were significant decreases (improvements) from week 0 at last observation and week 50 in all CAARS subscale scores (*p* <.0001; Table 4).

#### CGI-I, PGI-I, and CGI-S

The percentage of patients with “very much improved” or “much improved” physician-rated (CGI-I) and patient-rated (PGI-I) scores, and with “normal” or “borderline mentally ill” physician-rated CGI-S scores, increased during long-term GXR treatment (Table 4). Eighteen patients were rated as severely ill at week 0 (eight former placebo patients, four former GXR patients, and six new patients). At week 50, three were markedly ill (two former placebo patients, one new patient) and three remained severely ill (all former placebo patients), with the remainder rated as borderline, mildly, or moderately ill.

#### AAQoL and BRIEF-A

Patient-reported QoL and executive functioning significantly improved in former DBT patients who transitioned and in new patients during long-term treatment (Table 4). AAQoL total scores increased (improved) significantly from week 0 to 49.11 for former placebo patients, 58.27 for former GXR patients, and 52.39 for new patients at last observation. At last observation and week 50, significant improvements from week 0 were reported for AAQoL life productivity for former placebo and GXR patients, life outlook for new patients, and relationships for former placebo patients (Table 4). In addition, significant improvements were reported for almost all BRIEF-A T-score subscales in all populations (Table 4).

## Discussion

This is the first study to assess long-term safety and efficacy of dose-optimized GXR in adult ADHD. The safety findings during treatment for 50 weeks were consistent with the previous 10-week DBT [13] and the known safety profile of GXR, and no new or unexpected safety signals were identified. Adult patients experienced improvements in ADHD symptoms, QoL, and executive functioning that were sustained for up to 1 year. Given the complexity of treating ADHD, nonstimulant medication can be an important option for patients when other medications are not effective or well tolerated [6]. The findings from this study support the use of GXR as an alternative treatment for adult patients with ADHD in Japan.

Consistent with the known safety profile of GXR in children [24–27], the most frequently reported TEAEs were sedative and included somnolence, decreased blood pressure, thirst, postural dizziness, bradycardia, malaise, constipation, and dizziness. Although nasopharyngitis was reported frequently, this TEAE was not considered related to GXR. Similar to the previous DBT [13], thirst was reported more frequently in adults than in studies of GXR in children [28, 29]. This finding was not considered to be clinically relevant or related to any differences in ethnicity between Japanese and non-Japanese populations because thirst (dry mouth) has been reported in studies conducted with GXR in adults in the United States [30, 31] and because direct comparison of the pharmacokinetics, safety, and tolerability of GXR showed no major differences in safety profiles between healthy Japanese and adults in the United States [32]. In line with the decreases in blood pressure and heart rate that have been observed during treatment with GXR in children [25–27], eight patients discontinued because of mild to moderate reductions in blood pressure and four discontinued because of bradycardia; only one case of bradycardia was severe and the patient recovered after treatment discontinuation. One patient experienced the serious TEAE, supraventricular tachycardia, which was moderately severe and for which relatedness to GXR was not excluded. GXR is not known to affect cardiac repolarization [31], and there were no clinically relevant changes in cardiovascular parameters, vital signs, or body weight for patients who continued treatment for 50 weeks. There were no substantial differences in the proportion of patients experiencing TEAEs among the treatment populations. However, former GXR patients reported fewer treatment-related TEAEs, fewer TEAEs leading to discontinuation, and fewer TEAEs of moderate severity compared with former placebo patients and new patients (Table 2), which is to be expected given that most sedative events are transitory, occur within the first few weeks of treatment, and resolve over time [13, 26, 27].

Treatments that provide sustained long-term improvements in ADHD symptoms are needed for adults because of the substantial impact of ADHD in adults on general health, psychosocial and neuropsychological functioning, and productivity [9, 10, 33]. During the previous DBT, significant improvements in ADHD symptoms (ADHD-RS-IV total and subscale scores) compared with placebo were observed for GXR-treated patients at 4 weeks [13]. In the current study, rapid improvement in ADHD symptoms was seen for GXR-treated patients within the first 6 weeks, which continued to improve for up to 50 weeks. These improvements were similar to the improvements in patient-reported QoL and all aspects of executive functioning.

The main strength of this study is that the flexible-dosing regimen allowed individualized treatment in all patients for 50 weeks of treatment. Furthermore, multiple physician- and patient-specific rating instruments were included to assess the effects of treatment. Although all patients underwent titration at the start of the long-term treatment, patients who transitioned from GXR in the previous DBT did not undergo a washout phase and received continuous GXR treatment through to the end of long-term treatment. There was a potential for observer bias because of the open-label nature of the study, and the findings may not be representative of real-world settings because patients with psychiatric or cardiovascular comorbidities, which are common in patients with ADHD, were excluded. In addition, there was a potential bias favoring safety and efficacy for continuing patients because those who discontinued owing to adverse events or lack of efficacy were not eligible for inclusion. However, these effects are balanced by the inclusion of newly enrolled patients.

## Conclusions

In conclusion, there were no new or unexpected safety concerns during long-term administration of GXR in Japanese adults with ADHD. During long-term treatment for up to 50 weeks, patients who received dose-optimized GXR had improvements in multiple aspects of ADHD, including symptoms, QoL, and executive functioning.

## Supplementary information

**Additional file 1.**

## Data Availability

Researchers can request access to detailed information about Shionogi’s clinical trials, including trial protocols and individual patient data, on the portal site: clinicalstudydatarequest.com. Sharable information includes data about Shionogi’s clinical trials conducted in patients in Japan. The information will become sharable after the medicinal products for which the trials are performed have been approved in Japan. Note that all documents will be provided in Japanese language only as they have been prepared in Japanese.

## Abbreviations

AAQoL: Adult ADHD Quality of Life Questionnaire
ADHD: Attention-deficit/hyperactivity disorder
ADHD-RS-IV: ADHD Rating Scale IV with Adult Prompts
BRIEF-A: Behavior Rating Inventory of Executive Function-Adult Version
CAARS: Conners’ Adult ADHD Rating Scales
CGI-S: Clinical Global Impression-Severity of Illness scale
CI: Confidence interval
DBT: Double-blind trial
*DSM-5*: *Diagnostic and Statistical Manual of Mental Disorders* (Fifth Edition)
ECG: Electrocardiogram
GXR: Guanfacine extended-release
PGI-I: Patient Global Impression-Improvement scale
TEAEs: Treatment-emergent adverse events
QoL: Quality of life
