# Correction to: Safety and efficacy of guanfacine extended-release in adults with attention-deficit/hyperactivity disorder: an open-label, long-term, phase 3 extension study

**DOI:** 10.1186/s12888-020-02965-7

**Published:** 2020-12-22

**Authors:** Akira Iwanami, Kazuhiko Saito, Masakazu Fujiwara, Daiki Okutsu, Hironobu Ichikawa

**Affiliations:** 1 Department of Psychiatry, Showa University School of Medicine, 6-11-11 Kita Karasuyama, Setagaya-ku, Tokyo, 157-8577 Japan; 2 Aiiku Counselling Office, Aiiku Research Institute, Imperial Gift Foundation Boshi-Aiiku-Kai, Tokyo, Japan; 3 Biostatistics Center, Shionogi & Co., Ltd., Osaka, Japan; 4 Clinical Research Department, Shionogi & Co., Ltd, Osaka, Japan; 5 Japan Developmental Disorders Network, Tokyo, Japan

**Correction to: BMC Psychiatry 20, 485 (2020)**

**https://doi.org/10.1186/s12888-020-02867-8**

Following publication of the original article [1], the authors identified an error in Table 4. The correct table is given below.

**Table 4:** Key Efficacy Measures During Long-term Treatment With GXR

			Week 50		Last observation in the treatment period	
Endpoint	Patient populations	Week 0	Change from week 0	*p*-value	Change from week 0	*p*-value
ADHD-RS-IV^a^, mean (95% CI)						
Total scores	Former placebo patients	24.76 (22.53, 26.99)	−8.31 (−10.72, −5.89)	<.0001	−5.94 (−7.53, −4.36)	<.0001
	Former GXR patients	22.31 (19.65, 24.97)	−9.11 (−11.19, −7.03)	<.0001	−7.87 (−9.68, −6.06)	<.0001
	New patients	32.80 (30.93, 34.68)	−19.69 (−23.35, −16.03)	<.0001	−16.54 (−19.77, −13.31)	<.0001
Inattention score	Former placebo patients	17.36 (15.97, 18.76)	−5.51 (−7.15, −3.87)	<.0001	−3.90 (−5.03, −2.76)	<.0001
	Former GXR patients	15.37 (13.68, 17.07)	−5.82 (−7.22, −4.42)	<.0001	−4.87 (−6.04, −3.70)	<.0001
	New patients	21.68 (20.12, 23.24)	−12.10 (−14.70, −9.51)	<.0001	−10.02 (−12.28, −7.76)	<.0001
Hyperactivity-impulsivity score	Former placebo patients	7.40 (6.15, 8.65)	−2.80 (−4.00, −1.59)	<.0001	−2.05 (−2.77, −1.32)	<.0001
	Former GXR patients	6.94 (5.55, 8.32)	−3.29 (−4.40, −2.17)	<.0001	−3.00 (−3.96, −2.04)	<.0001
	New patients	11.12 (9.50, 12.74)	−7.59 (−9.81, −5.36)	<.0001	−6.51 (−8.25, −4.78)	<.0001
CAARS scores (*DSM-IV*)^a^, mean (95% CI)						
Total ADHD symptoms	Former placebo patients	25.08 (22.93, 27.23)	−6.27 (−8.65, −3.89)	<.0001	−4.60 (−6.17, −3.02)	<.0001
	Former GXR patients	22.74 (20.07, 25.42)	−8.38 (−10.90, −5.86)	<.0001	−7.30 (−9.49, −5.10)	<.0001
	New patients	31.32 (28.64, 33.99)	−17.31 (−20.89, −13.73)	<.0001	−15.08 (−18.49, −11.66)	<.0001
Inattentive symptoms	Former placebo patients	17.40 (16.14, 18.65)	−3.96 (−5.57, −2.35)	<.0001	−2.90 (−4.02, −1.79)	<.0001
	Former GXR patients	15.55 (13.90, 17.19)	−5.40 (−7.03, −3.77)	<.0001	−4.51 (−5.89, −3.13)	<.0001
	New patients	20.39 (18.48, 22.30)	−11.00 (−13.54, −8.46)	<.0001	−9.15 (−11.47, −6.83)	<.0001
Hyperactive-impulsive symptoms	Former placebo patients	7.68 (6.38, 8.98)	−2.31 (−3.55, −1.06)	.0005	−1.69 (−2.47, −0.92)	<.0001
	Former GXR patients	7.19 (5.79, 8.60)	−2.98 (−4.30, −1.65)	<.0001	−2.79 (−3.92, −1.66)	<.0001
	New patients	10.93 (9.36, 12.49)	−6.31 (−8.17, −4.45)	<.0001	−5.93 (−7.56, −4.29)	<.0001
CGI-I response rates^b^, % of patients (95% CI)						
Improvement rate (disease scores 1 or 2)	Former placebo patients	3.4 (0.7, 9.6)^c^	51.0 (36.3, 65.6)	NA	35.2 (25.3, 46.1)	NA
	Former GXR patients	4.8 (1.0, 13.5)^c^	64.4 (48.8, 78.1)	NA	53.2 (40.1, 66.0)	NA
	New patients	0.0 (0.0, 8.6)^c^	79.3 (60.3, 92.0)	NA	65.9 (49.4, 79.9)	NA
PGI-I response rates^b^, % of patients (95% CI)						
Improvement rate (disease scores 1 or 2)	Former placebo patients	8.0 (3.3, 15.7)^c^	28.6 (16.6, 43.3)	NA	19.3 (11.7, 29.1)	NA
	Former GXR patients	9.7 (3.6, 19.9)^c^	42.2 (27.7, 57.8)	NA	33.9 (22.3, 47.0)	NA
	New patients	9.8 (2.7, 23.1)^c^	37.9 (20.7, 57.5)	NA	31.7 (18.1, 48.1)	NA
Patients not ill or borderline mentally ill^b^, % of patients (95% CI)						
CGI-S scores 1 or 2	Former placebo patients	0.0 (0.0, 4.1)	14.3 (5.9, 27.2)	NA	8.0 (3.3, 15.7)	NA
	Former GXR patients	0.0 (0.0, 5.8)	26.7 (14.6, 41.9)	NA	22.6 (12.9, 35.0)	NA
	New patients	0.0 (0.0, 8.6)	20.7 (8.0, 39.7)	NA	17.1 (7.2, 32.1)	NA
AAQoL^a^, mean (95% CI)						
Total score	Former placebo patients	46.43 (43.21, 49.64)	4.13 (0.50, 7.75)	.0266	2.81 (0.31, 5.30)	.0282
	Former GXR patients	54.27 (49.78, 58.77)	4.29 (0.35, 8.23)	.0334	4.04 (0.88, 7.20)	.0131
	New patients	43.28 (38.38, 48.17)	12.75 (6.68, 18.81)	.0002	9.22 (4.11, 14.34)	.0008
Life productivity	Former placebo patients	48.04 (43.75, 52.33)	2.64 (−3.32, 8.61)	.3775	2.89 (−0.94, 6.72)	.1377
	Former GXR patients	57.88 (52.69, 63.08)	8.74 (4.69, 12.79)	<.0001	8.08 (4.76, 11.41)	<.0001
	New patients	44.29 (37.72, 50.86)	17.08 (9.11, 25.06)	.0001	14.38 (7.75, 21.00)	<.0001
Psychological health	Former placebo patients	47.02 (42.20, 51.83)	5.27 (0.57, 9.97)	.0286	2.60 (−1.14, 6.34)	.1710
	Former GXR patients	54.91 (48.88, 60.93)	2.78 (−2.69, 8.25)	.3117	1.57 (−2.82, 5.96)	.4771
	New patients	43.39 (36.92, 49.86)	11.35 (4.29, 18.41)	.0027	5.62 (−0.57, 11.82)	.0739
Life outlook	Former placebo patients	41.93 (38.20, 45.66)	2.59 (−1.97, 7.15)	.2597	1.37 (−1.76, 4.51)	.3868
	Former GXR patients	46.10 (41.64, 50.56)	−1.90 (−6.44, 2.63)	.4016	−0.35 (−4.08, 3.37)	.8510
	New patients	40.17 (35.10, 45.24)	8.23 (1.25, 15.21)	.0225	6.06 (0.71, 11.41)	.0275
Relationships	Former placebo patients	48.47 (44.01, 52.92)	8.16 (2.70, 13.63)	.0042	4.88 (1.15, 8.61)	.0109
	Former GXR patients	57.02 (50.97, 63.06)	5.00 (−1.10, 11.10)	.1058	4.26 (−0.82, 9.34)	.0984
	New patients	45.24 (38.72, 51.77)	11.21 (3.26, 19.16)	.0074	6.63 (−0.12, 13.37)	.0542
BRIEF-A T-score^a^, mean (95% CI)						
Inhibit	Former placebo patients	57.24 (54.81, 59.66)	−3.69 (−6.39, −0.99)	.0084	−2.39 (−4.15, −0.63)	.0084
	Former GXR patients	51.68 (49.20, 54.16)	−1.84 (−4.04, 0.35)	.0977	−2.66 (−4.65, −0.66)	.0098
	New patients	59.68 (56.49, 62.87)	−8.07 (−11.76, −4.38)	.0001	−8.25 (−11.28, −5.22)	<.0001
Shift	Former placebo patients	69.55 (66.83, 72.27)	−5.84 (−9.00, −2.68)	.0005	−3.29 (−5.51, −1.08)	.0040
	Former GXR patients	62.73 (59.04, 66.41)	−4.60 (−8.30, −0.90)	.0159	−3.70 (−6.57, −0.84)	.0121
	New patients	70.07 (66.33, 73.81)	−8.86 (−11.81, −5.91)	<.0001	−8.63 (−11.18, −6.07)	<.0001
Emotional control	Former placebo patients	57.80 (55.51, 60.08)	−4.39 (−7.55, −1.23)	.0075	−3.26 (−5.34, −1.18)	.0025
	Former GXR patients	53.32 (50.75, 55.89)	−2.22 (−4.97, 0.53)	.1104	−1.52 (−3.72, 0.67)	.1703
	New patients	59.98 (56.74, 63.22)	−5.41 (−7.60, −3.23)	<.0001	−4.53 (−6.68, −2.37)	.0001
Self-monitor	Former placebo patients	61.81 (58.70, 64.91)	−6.39 (−8.98, −3.79)	<.0001	−4.48 (−6.45, −2.52)	<.0001
	Former GXR patients	56.06 (52.73, 59.40)	−4.93 (−8.10, −1.76)	.0031	−4.23 (−6.87, −1.58)	.0022
	New patients	61.24 (56.70, 65.79)	−7.86 (−11.77, −3.96)	.0003	−6.23 (−10.05, −2.40)	.0021
Behavioral regulation index	Former placebo patients	63.09 (60.46, 65.73)	−6.02 (−9.06, −2.98)	.0002	−4.06 (−6.05, −2.07)	.0001
	Former GXR patients	56.39 (53.43, 59.35)	−3.71 (−6.52, −0.90)	.0109	−3.26 (−5.52, −1.01)	.0053
	New patients	64.73 (61.24, 68.23)	−8.72 (−11.53, −5.92)	<.0001	−7.98 (−10.72, −5.23)	<.0001
Initiate	Former placebo patients	68.51 (65.65, 71.38)	−5.49 (−8.51, −2.46)	.0006	−3.87 (−5.94, −1.80)	.0004
	Former GXR patients	59.71 (56.58, 62.84)	−3.31 (−6.64, 0.02)	.0514	−2.03 (−4.87, 0.80)	.1563
	New patients	69.10 (64.97, 73.22)	−10.38 (−13.98, −6.78)	<.0001	−8.95 (−12.11, −5.79)	<.0001
Working memory	Former placebo patients	73.91 (71.07, 76.75)	−4.80 (−8.45, −1.15)	.0111	−3.31 (−5.68, −0.93)	.0069
	Former GXR patients	66.18 (62.57, 69.78)	−4.78 (−8.24, −1.32)	.0079	−3.92 (−6.70, −1.13)	.0066
	New patients	74.10 (70.29, 77.91)	−10.93 (−14.98, −6.88)	<.0001	−10.15 (−13.73, −6.57)	<.0001
Plan/organize	Former placebo patients	70.51 (67.74, 73.28)	−3.86 (−6.94, −0.78)	.0152	−2.32 (−4.37, −0.27)	.0270
	Former GXR patients	63.02 (59.52, 66.51)	−3.69 (−7.09, −0.29)	.0340	−3.00 (−5.90, −0.10)	.0426
	New patients	70.51 (66.57, 74.45)	−8.90 (−12.63, −5.16)	<.0001	−8.75 (−11.90, −5.60)	<.0001
Task monitor	Former placebo patients	72.63 (69.56, 75.69)	−6.76 (−9.95, −3.56)	<.0001	−4.07 (−6.35, −1.79)	.0006
	Former GXR patients	63.85 (60.22, 67.49)	−7.02 (−11.06, −2.99)	.0011	−4.89 (−8.34, −1.43)	.0063
	New patients	0.71 (66.07, 75.34)	−8.93 (−13.17, −4.70)	.0002	−8.35 (−12.12, −4.58)	<.0001
Organization of materials	Former placebo patients	65.97 (63.95, 67.98)	−5.00 (−7.82, −2.18)	.0008	−3.24 (−5.21, −1.26)	.0016
	Former GXR patients	58.61 (55.77, 61.45)	−3.31 (−5.55, −1.08)	.0046	−2.87 (−4.62, −1.11)	.0018
	New patients	65.73 (62.43, 69.03)	−8.41 (−11.90, −4.93)	<.0001	−7.88 (−11.03, −4.72)	<.0001
Metacognition index	Former placebo patients	73.36 (70.51, 76.21)	−5.80 (−9.04, −2.55)	.0008	−3.75 (−5.86, −1.65)	.0007
	Former GXR patients	64.16 (60.65, 67.68)	−5.02 (−8.21, −1.83)	.0028	−3.82 (−6.44, −1.20)	.0050
	New patients	73.24 (69.29, 77.20)	−11.14 (−14.95, −7.33)	<.0001	−10.35 (−13.69, −7.01)	<.0001
GEC index	Former placebo patients	70.52 (67.76, 73.29)	−6.41 (−9.59, −3.22)	.0002	−4.22 (−6.30, −2.15)	.0001
	Former GXR patients	61.73 (58.32, 65.13)	−4.84 (−7.96, −1.73)	.0031	−3.90 (−6.45, −1.36)	.0032
	New patients	71.10 (67.26, 74.93)	−10.86 (−14.29, −7.43)	<.0001	−10.05 (−13.21, −6.89)	<.0001

*AAQoL* Adult ADHD Quality of Life Questionnaire, *ADHD-RS-IV* Attention-Deficit/Hyperactivity Disorder Rating Scale IV with Adult Prompts, *BRIEF-A* Behavior Rating Inventory of Executive Function-Adult Version, *CAARS* Conners’ Adult ADHD Rating Scales, *CGI-I* Clinical Global Impression-Improvement, *CGI-S* Clinical Global Impression-Severity of Illness, *CI* confidence interval, *DSM-IV* Diagnostic and Statistical Manual of Mental Disorders (Fourth Edition), *GEC* Global Executive Composite, *GXR* guanfacine extended-release, *NA* not applicable, *PGI-I* Patient Global Impression-Improvement

^a^Change from start of long-term treatment calculated using week 50 or last observation in the treatment period and assessed using two-sided *t* tests

^b^Data are response rates at each time point. Differences in response rates from the start of long-term treatment or week 1 and week 50 or last observation in the treatment period were assessed using two-sided *t* tests

^c^Data are response rates at week 1 of long-term treatment

The author group has been updated above and the original article [1] has been corrected.
