# Supplementary material for: Safety and efficacy of guanfacine extended-release in adults with attention-deficit/hyperactivity disorder: an open-label, long-term, phase 3 extension study
Source: BMC Psychiatry. 2020 Oct 2;20:485. doi: 10.1186/s12888-020-02867-8 (PMC7531113; doi:10.1186/s12888-020-02867-8)

Supporting information

Safety and efficacy of guanfacine extended-release in adults with attention-deficit/hyperactivity disorder: an open-label, long-term, phase 3 extension study

Akira Iwanami, Kazuhiko Saito, Masakazu Fujiwara, Daiki Okutsu, Hironobu Ichikawa

Additional file 1 Study design

Patients in the previous DBT who completed 10 weeks of treatment and were undergoing tapering (week 11 of the DBT) were eligible for inclusion in the long-term treatment study and started at 2 mg/day GXR with no washout period. DBT, double-blind trial; GXR, guanfacine extended-release.

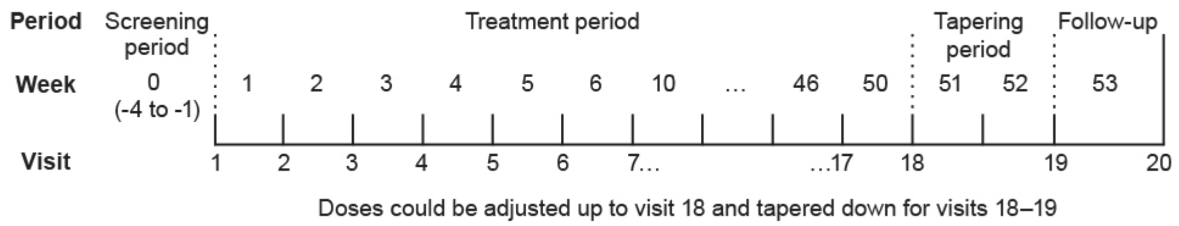

Supplement: Supplementary file 1 — Additional file 1. [file 12888_2020_2867_MOESM1_ESM.pdf]
